# Identification of Female Sex Pheromone of a Plant Bug, *Polymerus pekinensis* Reuter (Hemiptera: Miridae)

**DOI:** 10.3390/insects16020111

**Published:** 2025-01-23

**Authors:** Liuyang Wang, Yubo Wang, Xiaofang Zhang, Meijuan Fang, Xiangdong Mei, Tao Zhang

**Affiliations:** 1 Plant Protection Institute, HAAFS/Key Laboratory of IPM on Crops in Northern Region of North China, Ministry of Agriculture and Rural Affairs, China/IPM Innovation Center of Hebei Province/International Science and Technology Joint Research Center on IPM of Hebei Province, Baoding 071000, China; wliuyang1008@163.com (L.W.); zxfang224@163.com (X.Z.); 2 Dry-Land Farming Institute, Hebei Academy of Agricultural and Forestry Sciences, Hengshui 053000, China; wybnky@126.com (Y.W.); fangmeijuan@163.com (M.F.); 3 State Key Laboratory for Biology of Plant Diseases and Insect Pests, Institute of Plant Protection, Chinese Academy of Agricultural Sciences, Beijing 100193, China; xdmei@ippcaas.cn

**Keywords:** Miridae, chemical communication, octyl acetate, decyl acetate, trapping

## Abstract

Insect sex pheromones have been widely used in integrated pest control due to their efficiency, non-toxicity, specificity, and environmental sustainability. They are considered a key component of green pest management techniques. *Polymerus pekinensis* is a phytophagous plant bug on alfalfa (*Medicago sativa* L.) in East Asia. This study used gas chromatography–electroantennogram detection (GC–EAD) and gas chromatography–mass spectrometry (GC–MS) to analyze the whole-body extracts from male and female *P. pekinensis*. Octyl acetate (OA) and decyl acetate (DA) elicited the antennal response of males and were identified as the predominant components of female and male extracts, respectively. Subsequent field trials demonstrated that OA (>8 mg per lure) showed the strongest attraction to conspecific males. However, when DA was added in a lure (≥2 mg), a significant decline in captures occurred. These findings provide new insights into the understanding of sex pheromones in Miridae and benefit the development of sustainable management of *P. pekinensis*.

## Simple Summary

Plant bugs’ locating of mates mainly relies on peculiar chemical signals known as sex pheromones, which can be utilized to develop environmentally friendly pest control methods. This study investigated the sex pheromones of *Polymerus pekinensis*, a phytophagous insect of alfalfa crops in East Asia. By analyzing chemical extracts from both male and female insects, we identified two antenna-active compounds: octyl acetate (OA) and decyl acetate (DA). Females produced higher amounts of OA, while males produced greater quantities of DA. Field experiments demonstrated that lures loaded with solely OA strongly attracted male *P. pekinensis*, making it a valuable tool for monitoring this pest. However, the addition of DA in larger amounts significantly decreased the attractivity of lures. Our findings offer new strategies for monitoring and managing these insects in a manner that does not harm non-target insects.

## 1. Introduction

Chemical communication is vital for insect survival and reproduction, facilitating both intraspecific and interspecific interactions through sex pheromones [1,2]. Since the identification of the first insect sex pheromone from the silkworm [3], over 3000 sex pheromone compounds have been isolated and identified from more than 90 families across nine orders of insects [4]. Due to their high species specificity and environmental sustainability, sex pheromones play essential roles in integrated pest management (IPM), e.g., pest monitoring, mass trapping, mating disruption, and biological control [5,6]. The effectiveness of pheromone-based approaches has been demonstrated across various insect orders, particularly in Lepidoptera and Hemiptera, which have become integral components of IPM [1,5,7].

The Miridae is one of the largest families in the order Heteroptera, containing over 11,000 species [7,8]. Many mirids feed on plant sap and are recognized as agricultural pests [7]. Chemical communication between sexes plays a vital role in mirid reproduction and species isolation [9]. In recent years, female sex pheromones in over 30 species were revealed, including *Apolygus lucorum* [10], *Lygus pratensis* [11], *Sahlbergella singularis* [12], *Distantiella Theobroma* [12], *Helopeltis cinchonae* [13], *Apolygus spinolae* [14], *L. lineolaris* [15], *Lygocoris pabulinus* [16], and *L. rugulipennis* [17]. These pheromones are primarily composed of saturated or mono-unsaturated unbranched ester or aldehyde compounds [9,10,11,13]. Representative components include hexyl butyrate, (*E*)-2-hexenyl butyrate, (*E*)-4-oxo-2-hexenal, and (*E*)-2-octenyl butyrate [7], which are likely the candidate components of sex pheromones in other Miridae species [9,11,12]. The pheromone compounds are produced in varying secretion sites among species. Metathoracic scent glands are the most frequently reported organs in many mirids like *Adelphocoris suturalis* [18], *Phytocoris calli* [19], *A. spinolae* [20], and *Orthops Campestris* [20]. Identification and functional verification of these pheromonal chemicals have provided the foundation for developing species-specific monitoring and control strategies against mirids.

The plant bug, *Polymerus pekinensis* Horváth (Hemiptera: Miridae) (NCBI: txid1929840), is widely distributed in East Asia, including China, Korea, and Japan (http://museum.ioz.ac.cn, (accessed on 29 December 2024)) [21,22]. Besides the main host plants, alfalfa (*Medicago sativa* L.) and *Rubia cordifolia* L., it also feeds on *Phaseolus vulgaris* L., *Vicia faba* L., *Brassica napus*, and potato *Solanum tuberosum* L. [21,22]. Both nymphs and adults pierce and suck plant juices, causing extensive chlorosis and defoliation in alfalfa, consequently leading to substantial economic losses [9,21,22]. Current management of *P. pekinensis* and other mirid bugs heavily relies on broad-spectrum insecticides such as chlorpyrifos, malathion, and cyhalothrin [7,22]. However, this chemical-based approach raises serious environmental concerns, including soil and groundwater contamination, and poses risks to beneficial non-targets [23,24]. The development of pheromone-based monitoring and control methods has shown promise in various mirid species [1,10], offering more environmentally friendly alternatives [1,25]. However, the sex pheromone of the *Polymerus* genus remains unknown. Based on our preliminary observations in the field, *P. pekinensis* females exhibited typical “sex calling” behavior in the scotophase like *A. lucorum* [10], *L. pratensis* [11], and *Ph. calli* [19], suggesting that female-produced sex pheromones played vital roles in the mating communication of this plant bug.

This study aimed to identify the sex pheromone produced by female *P. pekinensis* using gas chromatography–electroantennogram detection (GC–EAD) and gas chromatography–mass spectrometry (GC–MS), as well as to assess their attractiveness to *P. pekinensis* in field conditions. Identification of sex pheromones in the *Polymerus* genus will enhance our understanding of the chemical communication of insects and provide a foundation for developing monitoring strategies for *P. pekinensis*.

## 2. Materials and Methods

### 2.1. Insects and Pheromone Collection

*P. pekinensis* nymphs were collected from the farm of the Institute of Plant Protection, Hebei Academy of Agricultural and Forestry Sciences (38.95° N, 115.45° E), Baoding, China. The nymphs were reared in boxes (18 cm × 12 cm × 7 cm) at 26 ± 2 °C, 60–70% relative humidity (RH), and a 16:8 photoperiod (L:D). They were fed with fresh pods of *Phaseolus vulgaris*, which were replaced every two days. After adult emergence, individuals were separated by sex and maintained in cages (30 cm × 30 cm × 30 cm). Virgin sexual maturity adults (3–5 days old) were used for sex pheromone collection and electrophysiologic experiments [10].

Pheromone collection was conducted using the whole-body extraction method [10,11]. Briefly, a virgin female or male was separately closed in a 2 mL centrifuge tube and allowed to acclimate in the dark for 2 h. Subsequently, 500 μL of n-Hexane was added to immerse the individuals for pheromone collection. After a 5 min immersion, the supernatant was transferred to a sample vial and stored at −20 °C until subsequent GC–EAD and GC–MS analysis. The quantities of OA and DA in the extracts were determined using the external standard method with standards.

### 2.2. Chemicals, Lures, and Traps

Octyl acetate (OA) and decyl acetate (DA) (purity > 99%) were purchased from Aladdin Reagent Co., Ltd. (Shanghai, China). Polyethylene (PE) vials (25 mm× 6 mm× 0.25 mm; Pherobio Technology Co., Ltd., Beijing, China) were used as pheromone dispensers in the field experiments. The lures for field trapping were prepared by dissolving pheromones in 200 μL of sunflower oil (food grade) containing 1.0% butylated hydroxytoluene (BHT) and loading it into the PE vials [11]. After pheromone loading, the vials were heat-sealed immediately, allowing the compounds to diffuse and release through the PE wall at a slow rate. Triangle-shaped traps with removable sticky boards (Pherobio Technology Co., Ltd., Beijing, China) were used in the field experiments.

### 2.3. GC–EAD Recording

Electrophysiologic analyses of female and male extracts were conducted on a gas chromatograph (GC2030; Shimadzu, Kyoto, Japan) equipped with a DB-WAX column (30 m × 0.25 mm × 0.25 μm film thickness; Agilent Technologies, Santa Clara, CA, USA) and a 1:1 effluent splitter that allowed for simultaneous flame ionization detection (FID) and electroantennogram detection (EAD). The EAD section consisted of two electrodes, a data acquisition interface board (IDAC-2) and a CS-55 air stimulus controller (Syntech Ltd., Buchenbach, Germany). The carrier gas was nitrogen, and the injector temperature was set at 230 °C. The GC oven was initiated at 50 °C (held 1 min) and then increased to 120 °C (5 °C/min, held 1 min), and it subsequently increased to 230 °C (10 °C/min, held 5 min). The transfer tube for EAG preparation was constantly heated at 200 °C, with the outlet being positioned within a humidified airstream (300 mL/min) directed over an antenna. The antennae (with tips removed) were connected to electrodes using micro-glass capillaries filled with a 0.9% saline solution. Antennal signals were recorded and analyzed using a computer equipped with an IDAC interface box and GC–EAD software (version 4.0; Syntech Ltd., Buchenbach, Germany). A total of 20 female and 20 male *P. pekinensis* specimens were utilized for the GC–EAD analysis.

### 2.4. GC–MS Analyses

The mirid extracts were analyzed on an Agilent 7890A GC coupled with a 5975C mass spectrometer (Agilent Technologies). The ionization voltage was 70 eV, and the ion source temperature was 230 °C. The analysis employed an identical column and oven program as described in the GC–EAD experiment. The mass spectra were compared with those reported in the NIST14 database, and the identification of pheromone components was conducted by comparing the GC retention times and mass spectra with those of authentic samples.

### 2.5. Electroantennogram (EAG) Recordings

EAG system and test protocols were performed as described above. All chemicals were tested at doses of 0.01, 0.1, 1, 10, 100, and 1000 μg. Each dose was delivered by applying 10 μL of the corresponding solution (prepared in n-hexane) to a filter paper strip (0.5 cm × 5 cm), which was then placed in a Pasteur pipette (15 cm long). n-hexane (10 μL) was used as a solvent control. The apparatus maintained two parallel airflows: a continuous flow and a stimulating flow, each set at 500 mL·min^−1^. Upon initiating recording with a pedal press, the compensatory airflow was automatically shut off, and the stimulating airflow passed over the filter paper strip for 0.1 s. An IDAC-2 stimulus controller (Syntech Ltd., Buchenbach, Germany) was used to measure and record the resulting antennal voltage changes. Each stimulus puff was followed by a solvent control puff, and the compounds were tested in increasing concentrations. The EAG values were corrected by subtracting the solvent response. For each compound, ten replicates for each sex were tested.

### 2.6. Field Trials

The field experiments were conducted in an abandoned peach orchard in Baoding, China (38.86° N, 115.59° E). This area had high plant coverage, with various weeds such as *Chenopodium album* and *Humulus scandens*, as well as *Rubia cordifolia*, the main host plant of *P. pekinensis*. Triangular traps were hung on peach branches placed 20 cm above the canopy of plants, with a minimum spacing of 10 m between traps. All field experiments used a completely randomized block design with three replicates. The captures on the sticky boards were recorded every three days. The traps with renewed sticky boards were randomly reassigned after each check.

Field experiment 1 (2–17 September 2022) was conducted to evaluate the attractiveness of antenna-active components identified through GC–EAD screening. Based on the quantitative GC–MS analysis of female extracts, the treatments included OA (5 mg), DA (50 μg), and a binary blend (OA: 5 mg + DA: 50 μg), as well as a negative control (200 μL sunflower oil) and a positive control (five live virgin females, 5VF). Virgin females and fresh *P. vulgaris* were placed in rearing tubes (height 4.5 cm, diameter 2 cm) (with gauze caps) as 5VF lures. The females and their food were replaced daily.

Field experiment 2 (20 September–5 October 2022) was performed to optimize the dose of the pheromone candidate. Different doses of OA (0.1, 0.4, 0.8, 1.6, 2, 4, 6, 8, 10, 15, and 20 mg) dissolved in 200 μL of sunflower oil with 1.0% BHT were loaded in each PE vial. The doses of lures were optimized by comparing the captures in traps baited with a series of OA doses. The positive and negative controls were the same as in experiment 1.

Field experiment 3 (7–22 October 2022) aimed to investigate the role of DA in attracting male *P. pekinensis*. Based on the results of field experiment 2, different doses of DA (0.1, 1, 2, 4, 6, and 8 mg) were added into the OA lures (8 mg) to evaluate their influence on trapping. Positive and negative controls were also conducted.

### 2.7. Statistical Analysis

The amounts of pheromone components (between female and male extracts) and the corrected EAG values (between OA and DA at the same concentration) were compared by independent-sample *t*-tests. The EAG values among series concentrations and capture data from the field experiments were analyzed using one-way ANOVA tests, followed by Tukey’s test. A significance level of 0.05 was applied for all analyses.

## 3. Results

### 3.1. Pheromone Identification

**Figure 1:**
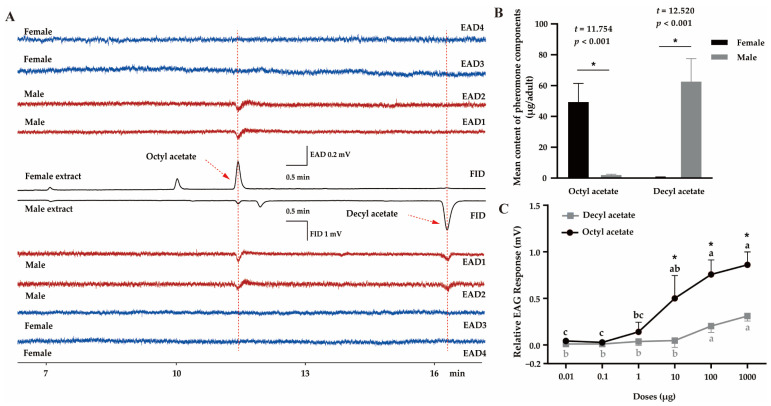
Identification of the putative sex pheromone of *Polymerus pekinensis*. (**A**) GC–EAD responses of male (red) and female (blue) antennae to female and male extracts. FID, flame ionization detector; EAD, electroantennogram detector. (**B**) The content of each component in female and male *P. pekinensis*, calculated according to authentic standards. Each value represents the mean of ten replicates. Significant differences are denoted by asterisks (*p* < 0.05). (**C**) EAG responses of *P. pekinensis* males (n = 10) to synthetic octyl acetate (OA) and decyl acetate (DA). For each compound, means with different letters are significantly different among doses by Tukey’s test (*p* < 0.05). Meanwhile, asterisks represent significant differences between OA and DA at the same concentration.

In the GC–EAD experiment, male antennae strongly responded to two compounds present in both female and male extracts, indicating that they were the potential candidates for sex pheromones (Figure 1). GC–MS results showed that the retention times and mass spectra of the two compounds highly corresponded with those of the authentic OA and DA, respectively (Figure 1A, Figures S1 and S2). In female extracts, OA was the major component (49.3 ± 12.1 μg per bug, *n* = 10), which was more abundant than that in the males (2.0 ± 0.6 μg per bug, *n* = 10) (*t* = 11.754, *p* < 0.001). In contrast, in males, the major component was DA (62.6 ± 14.9 μg, *n* = 10), which was significantly more than in the females (0.5 ± 0.2 μg, *n* = 10) (*t* = 12.520, *p* < 0.001) (Figure 1B). The GC–EAD results (Figure 1A) indicated that the contents of DA in female extracts and OA in male extracts were extremely low, with no response by male antennae. These findings predicted that the antennal response to OA and DA was associated with the chemical concentration. Further EAG results demonstrated that both OA and DA triggered significant dose-dependent EAG responses in male antennae (Figure 1C; OA: *F*_5,12_ = 21.334, *p* < 0.001; DA: *F*_5,12_ = 18.921, *p* < 0.001). However, OA elicited higher EAG responses than DA at the same dose, particularly 10 μg (*t* = 3.227, *p* = 0.032), 100 μg (*t* = 5.641, *p* = 0.005), and 1000 μg (*t* = 6.408, *p* = 0.003).

### 3.2. Field Trapping

**Figure 2:**
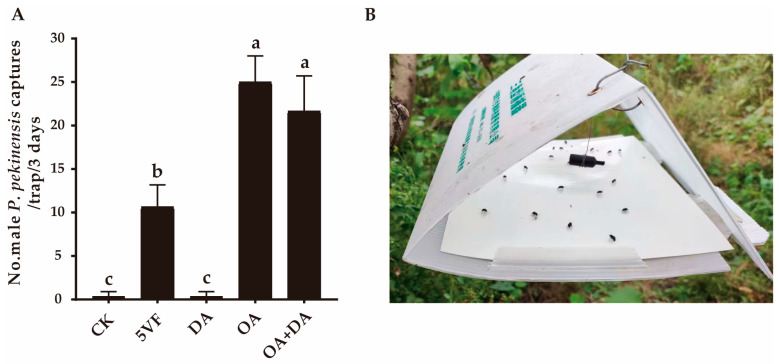
Attractiveness of potential pheromone components to *Polymerus pekinensis* males. (**A**) Captures of *Polymerus pekinensis* males in traps baited with the binary blend of octyl acetate (OA, 5 mg) and decyl acetate (DA, 50 μg) or each single component. CK, sunflower oil; 5VF, 5 virgin females. Different letters on each column represent significantly different results (*p* < 0.05). (**B**) Field attraction of triangle-shaped trap baited with a single-component OA lure.

Throughout the entire field experiment, no *P. pekinensis* females were caught in any traps. In field experiment 1, OA and the binary mixture of OA and DA attracted significantly more male *P. pekinensis*, compared with the trap with five virgin females (*F*_2,6_ = 15.989, *p* = 0.004) (Figure 2). No significant difference was found between them (*F*_1,4_ = 1.316, *p* = 0.315), indicating that OA was presumably the dominant contributor to chemical communication in *P. pekinensis*. This result was consistent with the finding that DA alone had minimal effect on attracting males (Figure 2). Thus, OA appeared to be the primary component of the sex pheromone in *P. pekinensis*.

**Figure 3:**
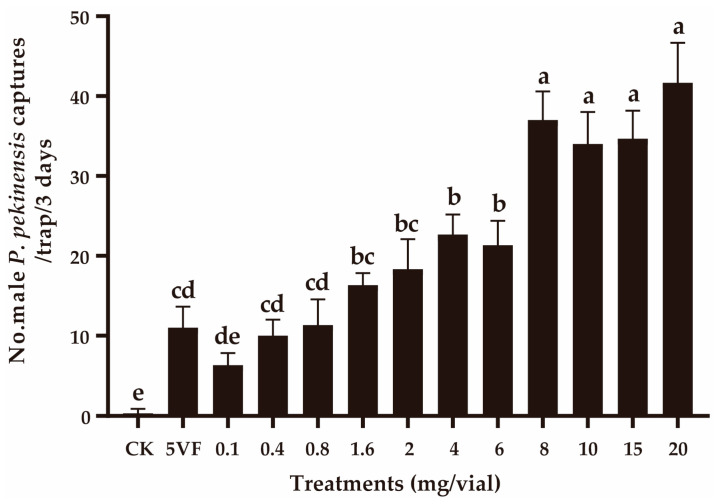
Captures of *Polymerus pekinensis* males in traps baited with varying doses of octyl acetate (OA). CK, sunflower oil; 5VF, 5 virgin females. Different letters represent significantly different results (*p* < 0.05).

In field experiment 2, the number of captures was positively related with the pheromone dose within the 0.8–8 mg addition of OA (Figure 3). When the pheromone doses were further increased, no significant change in capture numbers was observed (*F*_3,8_ = 2.167, *p* = 0.170) (Figure 3). At lower doses (0.1–0.8 mg), the capture numbers were similar to those obtained with traps containing five virgin female *P. pekinensis* (*F*_3,8_ = 2.667, *p* = 0.119).

**Figure 4:**
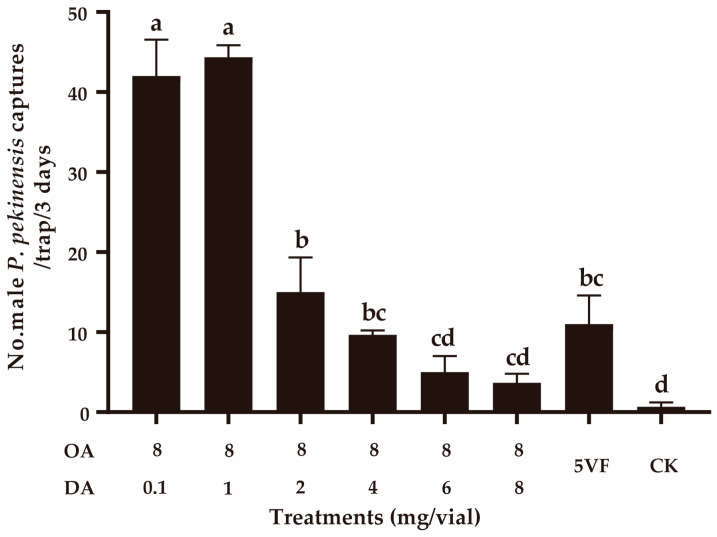
Effect of addition of decyl acetate (DA) on the attractiveness of octyl acetate (OA). CK, sunflower oil; 5VF, 5 virgin females. Different letters represent significantly different results (*p* < 0.05).

In field experiment 3, we confirmed that DA negatively functioned in the attractiveness by adding different doses (0.1–8 mg) of DA to the lure containing 8 mg of OA. With higher doses of DA being added (≥2 mg), the captures of OA lures significantly decreased (*F*_5,12_ = 127.474, *p* < 0.001) (Figure 4). When the OA:DA ratio was 1:1, capture numbers were not significantly different from those using the five virgin female *P. pekinensis* traps (*F*_1,4_ = 6.535, *p* = 0.065) (Figure 4). These results suggested that DA can function as a courtship inhibition pheromone in dosages higher than 1 mg.

## 4. Discussion

In the past decades, significant advancements have been made in the chemical ecology of mirids. For many mirids, sex attractants have demonstrated promising potential for monitoring and controlling mirid pests [9]. Notable examples include the identification and successful application of pheromones in managing *A. lucorum* [10], *L. pratensis* [11], *L. lineolaris* [15], *S. singularis* [12], and *L. rugulipennis* [26]. Through a comprehensive GC–EAD and GC–MS analyses of whole-body extracts from male and female *P. pekinensis*, we have successfully identified OA and DA as antenna-active components. Field trials have demonstrated that OA functioned as a primary attractant, while DA exhibited dose-dependent antagonistic effects. This study provides the first characterization of sex pheromone components in the *Polymerus* genus and provides new insight into the chemical communication of insects.

Traditionally, insect sex pheromone identification follows a stepwise approach: extraction of crude components, identification via GC–EAD and GC–MS analyses, behavioral screening (e.g., Y-tube olfactometer, cage tests, or wind tunnel experiments), and field trials [6,27]. In this study, we omitted the behavioral screening stage due to the specific behavioral characteristics of plant bugs. These insects require extended time to recover after mechanical disturbance [28]. Disturbed mirids commonly release sex pheromone and defensive compounds (such as hexyl butyrate, (*E*)-2-hexenal, and (*E*)-4-oxo-2-hexenal) [29,30] and exhibit abnormal locomotory patterns, e.g., rapid movements, excessive grooming, antenna waving, and wall-seeking behavior [9,31]. These traits will compromise the reliability of laboratory-based behavioral assays.

Although OA is detected in the extracts of several mirid species, such as *Ph. calli* [19] and *Ph. relativus* [32], it has not been identified as a sex pheromone component of plant bugs so far. Additionally, OA is also identified as an alarm pheromone component, herbivore-induced plant volatile (HIPV), or food-associated attractant that mimics fermented fruit volatiles [33,34,35]. The present study reported that OA solely served as the sex pheromone of *P. pekinensis*, which raises an intriguing concern: this plant bug might be attracted or confused by other organisms that contain and release OA. Actually, *P. pekinensis* successfully survives and flourishes with no disruption in chemical communication system by other organisms. An explanation is that the attractiveness of OA is easily affected or impaired by its analogs, like DA (Figure 4). In other mirids, OA is commonly detected as one component of gland secrets, while other components are its homologs or analogs. Taking *Ph. calli* for instance, the main female-specific components consist of hexyl acetate, (*E*)-2-hexenyl acetate, OA, and (*E*)-2-octenyl acetate, but the sex pheromone is identified as blend of hexyl acetate and (*E*)-2-octenyl acetate [19]. In the case of *Ph. calli* and *P. pekinensis* populations occurring in the same ecological niche, the large amount of hexyl acetate, (*E*)-2-hexenyl acetate, and (*E*)-2-octenyl acetate in *Ph. calli* females would contribute to preventing the attraction of *P. pekinensis* males. Similarly, (*E*)-2-octenyl acetate, an analog of DA, serves as an essential attractant component for *A. lucorum* and *Taylorilygus apicalis* males, while it strongly inhibits male attraction in other mirids, e.g., *A. spinolae*, *O campestris*, and *Stenotus rubrovittatus* [20]. Another reason that chemical communication of *P. pekinensis* is hardly disrupted by other OA-released organisms is that the OA amounts in females are relatively large for an insect, up to 49.3 ± 12.1 μg per individual (Figure 1B). OA from plant or fermented sources, as a comparison, is no more than 5 μg [34,35], and such trace amounts are unlikely to induce an electrophysiological (Figure 1) and behavioral (Figure 3) response of *P. pekinensis* males.

Many sex pheromone components are concurrently present in both females and males in several Miridae species [9], such as *Ph. calli* [19], *A. spinolae* [15], *A. suturalis* [36], and *Ph. relativus* [32]. Similarly, in *P. pekinensis*, OA and DA were detected in both sexes (Figure 1). While some pheromone compounds originate from males’ transfer during mating [37], the analyses of virgin adults in our study eliminate this possibility. Beyond sexual attraction, these compounds likely serve multiple ecological functions in Miridae, including aggregation, chemical defense, alarm signaling, and interspecific interactions [9]. Furthermore, certain pheromone components function as attractants of natural enemy behavior and inhibitions of entomopathogenic pathogen, suggesting an evolutionary advantage in insect survival [38,39]. This multifunctional nature of pheromone components may explain their presence in both sexes and reflect their broader ecological significance beyond reproductive communication [40].

The effectiveness of OA as a single-component attractant in *P. pekinensis* differs from the typical multi-component pheromone systems reported in most Miridae species [9,10,11,14,41]. For example, *H. cinchonae* uses a blend of 1-acetoxy-5-butyroxyhexane and hexyl 3-acetoxybutyrate in specific ratios [10], while *L. pratensis* employs hexyl butyrate, (*E*)-2-hexenyl butyrate, and (*E*)-4-oxo-2-hexenal for mate attraction [11]. Among *Apolygus* species, *A. lucorum* and *A. spinolae* share common components like (*E*)-4-oxo-2-hexenal, (*E*)-2-hexenyl butyrate, and hexyl butyrate but respond to different ratios, which contributes to reproductive isolation [15,20]. While multi-component systems often provide greater species specificity through unique compound ratios [5], our field experiment identified OA as the sole attractant in *P. pekinensis*. Interestingly, our results showed that DA did not exhibit antagonistic effects at lower dosages (0.1 and 1 mg per lure), suggesting that it had more nuanced roles beyond pheromone antagonism. This pattern parallels several well-documented cases in Lepidoptera, such as in *Macdunnoughia crassisigna*, where a blend of (*Z*)-7-dodecene acetate (Z7-12:OAc) and (*Z*)-9-tetradecene acetate (Z9-14:OAc) serves as the primary attractant at a 3:1 ratio, while (*Z*)-11-hexadecen-1-ol (Z11-16:OH) may contribute to species specificity [42]. Similarly, in *Amyelois transitella*, a four-component blend at a specific ratio functioned as the main sex attractant, while some minor components identified from female pheromone glands showed no effect on attraction [43]. From a practical perspective, a single-component system could streamline monitoring procedures and reduce synthesis costs for pest management [44]. However, the use of a single compound raises concerns about species specificity, particularly in areas where multiple mirid species coexist [9,20]. During our field trials, we did not trap other mirid species, which could be mainly due to the absence of other species with shared pheromone components during the study period [42], the species-specific feature of OA for *P. pekinensis*, or the presence of additional compounds or ecological factors that we have not yet identified [2,5,9].

Beyond the role of OA as an attractant, our study revealed an antagonistic effect of DA on pheromone response. In this study, despite showing lower antennal activity than OA, DA significantly weakened pheromone effectiveness, especially in higher concentrations. This inhibitory pattern mirrors observations in other mirid species through various mechanisms. For example, male-specific compounds in *A. lucorum* compete with attractants at antennal receptor sites [45], while in *L. hesperus*, male-produced compounds reduce female attractiveness after mating [16,46]. In *P. pekinensis*, a higher abundance of DA in males than females, combined with its antagonistic effects, suggested its potential role as an anti-pheromone [10,47].

The application potential of pheromone-based management for *P. pekinensis* warrants careful consideration. Although our traps efficiently attracted males, the impact on population dynamics still necessitates further investigation. As observed in other species, residual males may continue to achieve sufficient mating frequencies, thereby sustaining the population [48]. Future research should focus on the influence of pheromone attractants on the population dynamics of *P. pekinensis* and other mirids, the negative effects on natural enemies of mirids, and the optimization of application parameters to refine management strategies.

## 5. Conclusions

This study reported the first identification of sex pheromone components in the *Polymerus* genus. Based on the results of electrophysiology and field experiments, OA was identified as the sex pheromone of *P. pekinensis*, while DA was considered as an antagonist. The potential for using these compounds in monitoring systems and mating disruption strategies offers promising alternatives for sustainable *P. pekinensis* monitoring.

## Data Availability

The data presented in this study are available on request from the corresponding author.

## Supplementary Materials

The following supporting information can be downloaded at https://www.mdpi.com/article/10.3390/insects16020111/s1, Figure S1: Mass spectra of octyl acetate (OA). A shows the standards of OA, while B displays the *Polymerus pekinensis* extract of OA; Figure S2: Mass spectra of decyl acetate (DA). A shows the standards of DA, while B displays the *Polymerus pekinensis* extract of DA.
