# Supplementary material for: Identification of Female Sex Pheromone of a Plant Bug, Polymerus pekinensis Reuter (Hemiptera: Miridae)
Source: Insects. 2025 Jan 23;16(2):111. doi: 10.3390/insects16020111 (PMC11855770; doi:10.3390/insects16020111)
Supplement: Supplementary file 1 [file insects-16-00111-s001.zip › insects-3427990-supplementary.pdf]

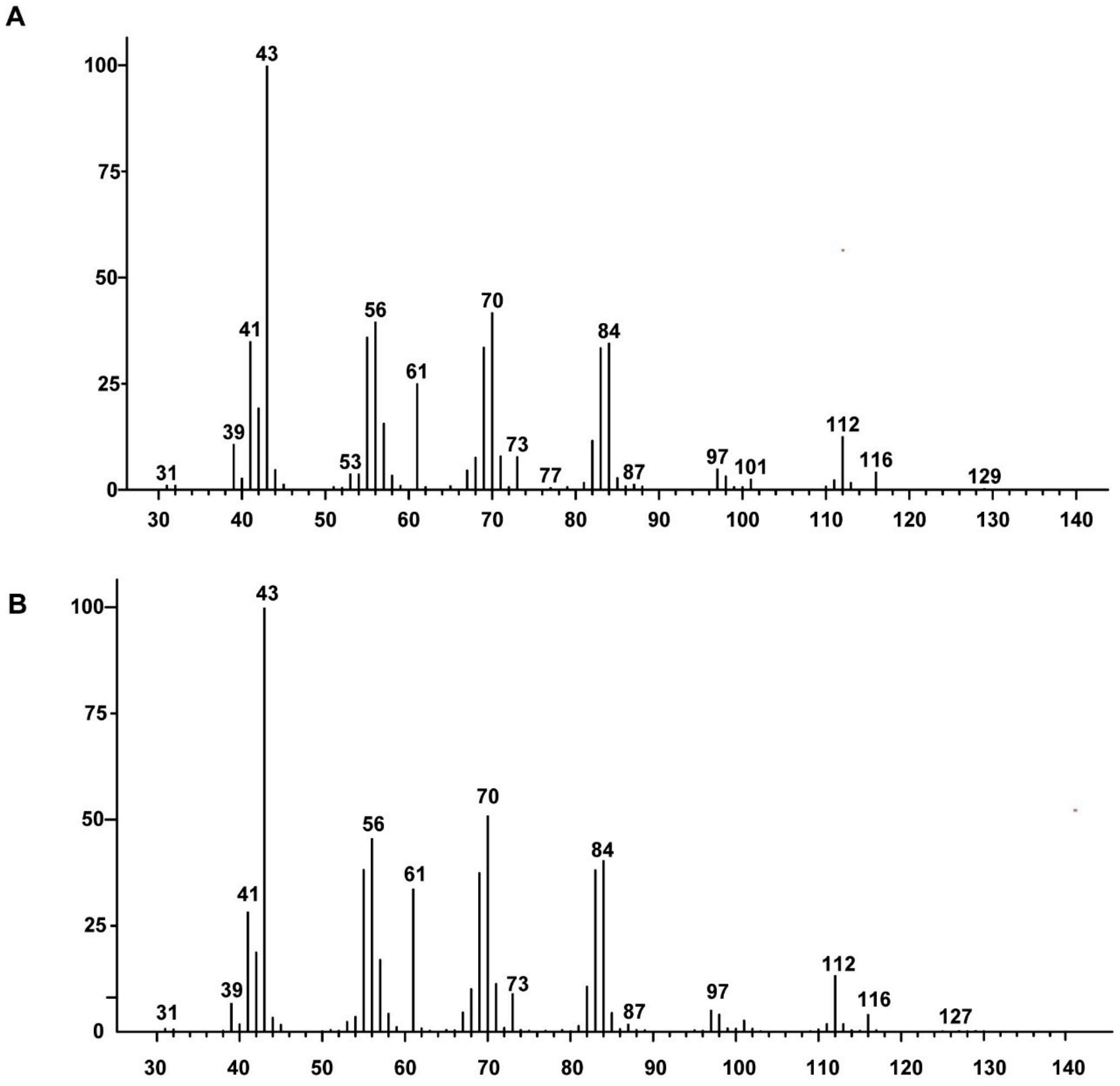

Figure S1. Mass spectra of octyl acetate (OA). A show the standards of OA, while B display the *Polymerus pekinensis* extract of OA.

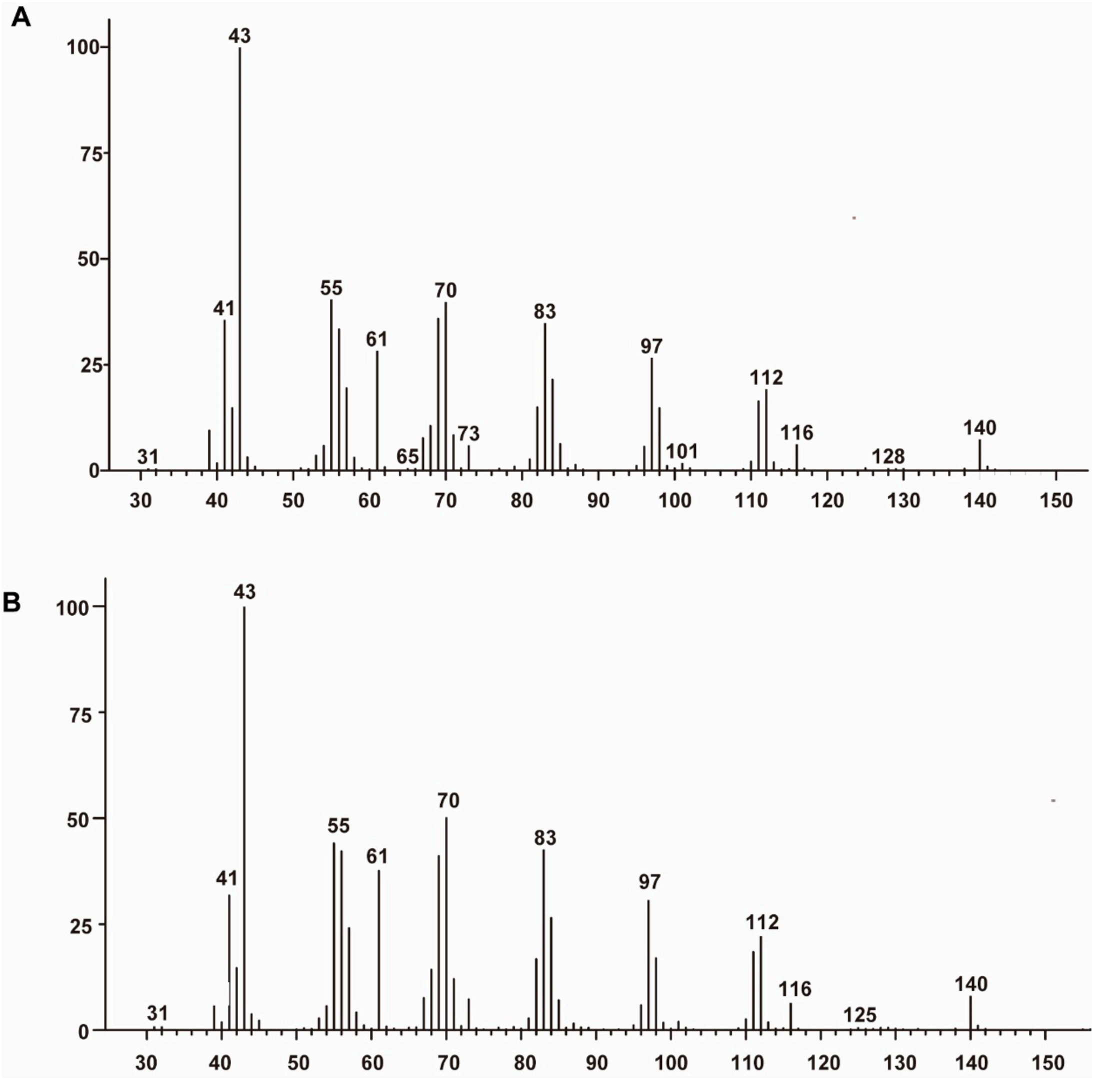

Figure S2. Mass spectra of decyl acetate (DA). A show the standards of DA, while B display the *Polymerus pekinensis* extract of DA.
